# Live SARS‐CoV‐2 is difficult to detect in patient aerosols

**DOI:** 10.1111/irv.12860

**Published:** 2021-05-03

**Authors:** Emily R. Robie, Anfal Abdelgadir, Raquel A. Binder, Gregory C. Gray

**Affiliations:** ^1^ Division of Infectious Diseases School of Medicine Duke University Durham NC USA; ^2^ Duke Global Health Institute Duke University Durham NC USA; ^3^ Global Health Research Center Duke Kunshan University Kunshan China; ^4^ Program in Emerging Infectious Diseases Duke‐NUS Medical School Singapore Singapore

## 1 DEAR EDITOR COWLING

As the COVID‐19 pandemic rages, there has been much debate regarding the importance of bioaerosols in SARS‐CoV‐2 transmission. Circumstantial evidence indicates that aerosol transmission is a likely contributor to the current pandemic,^1^, ^2^, ^3^ yet research teams have had difficulty isolating live virus when using traditional aerosol sampling techniques.^4^, ^5^, ^6^ To our knowledge, thus far only two research teams have successfully cultured airborne SARS‐CoV‐2 outside of laboratory simulations,^7^ with one team finding evidence of viral replication in the absence of cytopathic effect.^8^, ^9^

Despite the demonstrated challenges in capturing suspended live virus, respiratory transmission is now thought of as the primary mode of SARS‐CoV‐2 infection. By contrast, SARS‐CoV‐2 has been readily cultured from nasopharyngeal (NP) swabs, saliva, blood, stool, and semen.^4^, ^10^, ^11^, ^12^ Mounting evidence indicates that COVID‐19 patients are most infectious within the first eight days following symptom onset,^4^, ^12^, ^13^ with some outliers shedding live virus for up to 18 days.^13^, ^14^ Increased viral load is associated with better odds of culturing virus, with cut‐off values reported at 24 and 34 RT‐PCR cycle thresholds.^4^, ^13^

Building on previous work carried out by our team,^15^ we sought to enroll home isolated SARS‐CoV‐2 positive patients early in their disease progression to estimate the viability of the virus in biological, environmental, and bioaerosol samples and assess aerosol transmission of SARS‐CoV‐2.

From October 2020 to January 2021, we visited eight patients in their homes in and around Durham, North Carolina soon after they were confirmed by molecular assay to be infected with SARS‐CoV‐2. After informed consent was obtained, we asked patients to complete a brief questionnaire, and to permit the collection of a NP swab, passive saliva sample, fomite swabs, and bioaerosol samples. Patients were also asked to self‐collect a rectal swab sample. All study procedures were approved by the Duke University Institutional Review Board (Pro00105055).

Bioaerosol sampling was carried out using National Institute for Occupational Safety and Health (NIOSH) BC 251 aerosol samplers, placed ~1.5 meters off the ground at distances of ~1 meter, 1.4 meters, 2.2 meters, and 3.2 meters from the participant's head. The participants were asked to remain stationary in their room and the samplers were run for approximately two hours at a calibrated flow‐rate of 3.5 L_air_/min.^15^ SKC 20‐ml BioSamplers (SKC, Inc., Pennsylvania, USA), prepared with 16 mL phosphate‐buffered saline (PBS) and 0.5% bovine serum albumen (BSA), were placed beneath the NIOSH samplers on one side of the room and run simultaneously at the recommended flow rate of 12.5 L_air_/min. These samplers are designed to capture viral matter in liquid media to enhance viability. It is important to note that we have previously used both types of samplers to capture live influenza A virus.^16^, ^17^, ^18^, ^19^

Biological samples and fomite swabs were collected and processed as previously described,^15^ using FLOQSwab® (Copan, Murrieta, California) or sterile BD™ swabs (BD Diagnostics, Sparks, Maryland) and 1.5 mL VTM (Redoxica, Little Rock, Arkansas). Cell phones, TV remote controls, and door knobs were preferentially sampled, along with up to three other high‐touch surfaces, as indicated by the participant.

Viral RNA was extracted from processed samples using QIAamp Viral RNA Mini Kits (QIAGEN, Hilden, Germany), with the resultant product used in an adapted Center for Disease Control and Prevention (CDC) 2019‐nCoV real‐time RT‐PCR assay.^15^ Specimens with molecular evidence of SARS‐CoV‐2 infection were inoculated onto VeroE6/TMPRSS2 cells^20^ using 250 µl of sample for 7 days. Cells were monitored for cytopathic effect (CPE) every 48 hours. Cells and supernatant were harvested 7 days post‐inoculation. RNA extracts were then screened for SARS‐CoV‐2 with the real‐time RT‐PCR assay and considered positive when the CT value was at least 2 points below the original result and CPE was present.^15^

All participants presented with mild to moderate illness. The majority of the participants were females (n = 7, 87.5%). The predominantly represented race was white (n = 5, 62.5%), with two Black participants (25.0%), and one Asian (12.5%). One participant identified as Hispanic, and a second as Indian. The mean age was 41.4 years, with a range of 29 to 53 years. Among all participants, five lived in a private house, one in an apartment complex, and two in a residential shelter.

Participants were typically enrolled within three days of symptom onset (one patient was enrolled on day 8), and reported experiencing between zero and seven common COVID‐19 symptoms at time of enrollment, with cough, fatigue, and body ache the most frequently listed (Table S1). Chronic health conditions were reported by five participants, including hypertension, diabetes, and hypothyroidism. Three participants reported having traveled either domestically or internationally in the month prior to study enrollment.

Sampling sites in participants’ homes varied in size, with some permitting as few as 6 total bioaerosol samplers and others as many as 10 samplers. Among the eight participants, a total of 139 samples were collected. Among those, 45 (32.4%) were SARS‐CoV‐2‐positive by RT‐PCR, including 20 biological samples (83.3% of 24 total), 12 fomites (25.0% of 48 total), and 13 bioaerosol samplers (19.0% of 42 NIOSH Samplers, 20.0% of 25 SKC Samplers) (Table 1). Viable virus was recovered from six NP swabs (75.0%), five saliva samples (62.5%), and one rectal swab (12.5%). Despite this indicator of live viral shedding, none of the RT‐PCR positive fomite or bioaerosol samples had evidence of culturable SARS‐CoV‐2.

**TABLE 1:** Cycle threshold values for all samples with molecular evidence of SARS‐CoV‐2 nucleic acid, through the CDC 2019‐nCoV RT‐PCR assay. Samples with molecular evidence of SARS‐CoV‐2 were cultured in TMPRSS2 cells and considered positive upon observation of cytopathic effect followed by confirmatory RT‐PCR. Two forms of aerosol sampling (NIOSH BC 251 Cyclone Sampler and SKC 20 ml BioSampler) were simultaneously employed at varying distances from the participant's head

Patient	Sample Type^a^	N1 (Ct) ^b^	N2 (Ct) ^b^	Culture Results^c^	Fomites	Ct Range for N1 and N2^b^	Culture Results^c^	**Aerosol Samples**	**Ct Range** for N1 and N2^b^	**Culture Results** ^c^
1	Saliva	29.0	37.1	N	‐‐	‐‐	‐‐	‐‐	‐‐	‐‐
2	Saliva	38.2	39.0	N	‐‐	‐‐	‐‐	‐‐	‐‐	‐‐
3	NP Swab	16.9	17.3	P	Cell phone, sink handle (bathroom)	29.0 ‐ 39.0	N	1 m ‐ SKC; 1.4 m ‐ NIOSH	36.1 ‐ 38.6	N
	Saliva	29.1	33.7	P						
	Rectal Swab	35.0	34.6	N						
4	NP Swab	17.3	18.7	P	Cell phone, TV remote, bathroom door knob	33.4 ‐ 39.4	N	1.4 m ‐ SKC; 2.2 m ‐ SKC	36.4 ‐ 39.8	N
	Saliva	18.2	19.3	P						
	Rectal Swab	35.3	37.5	N						
5	NP Swab	17.9	18.6	P	Cell phone	37.8 ‐ 38.7	N	1.4 m ‐ NIOSH	36.9	N
	Saliva	29.5	31.6	P						
	Rectal Swab	34.1	37.0	N						
6	NP Swab	15.0	15.9	P	Cell phone, bathroom door knob, computer	36.7 ‐ 39.6	N	1 m ‐ NIOSH; 1.4 m ‐ NIOSH, SKC; 2.2 m ‐ NIOSH, SKC	31.8 ‐ 39.9	N
	Saliva	24.4	27.3	P						
	Rectal Swab	31.3	33.5	N						
7	NP Swab	18.1	18.6	P	Toilet handle	36.7 ‐ 38.6	N	‐‐	‐‐	‐‐
	Saliva	24.7	25.5	P						
	Rectal Swab	29.6	31.2	P						
8	NP Swab	25.6	26.7	P	TV remote, computer (mouse)	27.0 ‐ 39.3	N	‐‐	‐‐	‐‐
	Saliva	31.2	32.4	N						
	Rectal Swab	39.8	39.7	N						

^a^ NP=nasopharyngeal;

^b^ Ct=cycle threshold;

^c^ P=positive and N=negative =the patient's spouse (also SARS‐CoV‐2 positive) was present during sampling.

Findings presented here may not be representative of the general population, as recruitment was influenced by patient willingness to allow researchers into their homes and logistical constraints required households to be within close proximity of Duke University. Additionally, considerable variation between individual home floor plans affected the set‐up of aerosol samplers in participants’ living quarters.

Despite these limitations, this study importantly adds to the body of work demonstrating SARS‐CoV‐2 viability in varying biological samples gathered early in the disease progression of mild to moderate COVID‐19 illness, and affirms that fomites are unlikely to be a primary source of viral transmission. As compared to our previous efforts to capture live SARS‐CoV‐2 in bioaerosols,^15^ the use of VeroE6/TMPRSS2 cells (which are more sensitive in culturing SARS‐CoV‐2^20^ ) and the inclusion of the SKC BioSampler wet sampling technique (also thought to increase live virus detections) did not improve study findings. Our inability to detect viable virus in the air might be explained by insensitive sampling techniques or the notion that the participants had ceased shedding virus in aerosol by the time we engaged them.

## 2 ETHICS APPROVAL

All study procedures were approved by the Duke University Institutional Review Board (Pro00105055).

## CONFLICT OF INTEREST

None declared.

## AUTHOR CONTRIBUTION

**Emily R. Robie:** Data curation (lead); Investigation (equal); Project administration (supporting); Writing‐original draft (lead); Writing‐review & editing (equal). **Anfal Abdelgadir:** Investigation (equal); Writing‐review & editing (equal). **Raquel A. Binder:** Investigation (equal); Supervision (supporting); Writing‐review & editing (equal). **Gregory C**. **Gray:** Conceptualization (lead); Funding acquisition (lead); Investigation (equal); Methodology (lead); Project administration (lead); Resources (lead); Supervision (lead); Writing‐review & editing (equal).

### PEER REVIEW

The peer review history for this article is available at https://publons.com/publon/10.1111/irv.12860.

## Supporting information

Table S1

## Data Availability

The data that support the findings of this study are available from the corresponding author upon reasonable request.
