# Supplementary material for: Live SARS‐CoV‐2 is difficult to detect in patient aerosols
Source: Influenza Other Respir Viruses. 2021 May 3;15(4):554–7. doi: 10.1111/irv.12860 (PMC8189214; doi:10.1111/irv.12860)
Supplement: Supplementary file 1 — Table S1 [file IRV-15-554-s001.docx]

**Supplemental Information**

Live SARS-CoV-2 is Difficult to Detect in Patient Aerosols

Emily R. Robie,^1,2^ Anfal Abdelgadir,^1,2^

Raquel A. Binder,^1,2^ and Gregory C. Gray,^1,2,3,4^

**Affiliations:**

^1^Division of Infectious Diseases, School of Medicine, Duke University, Durham, North Carolina, USA

^2^Duke Global Health Institute, Duke University, Durham, North Carolina, USA

^3^Global Health Research Center, Duke Kunshan University, Kunshan, Jiangsu, China

^4^Program in Emerging Infectious Diseases, Duke-NUS Medical School, Singapore

**Corresponding author:** Gregory C. Gray, MD, MPH, FIDSA, DUMC Box 102359,

Durham, NC 27710, USA, Tel: +1-919-684-1032, Email: [gregory.gray@duke.edu](mailto:gregory.gray@duke.edu)

| **Supplemental Table 1.** Participant presentation and potential COVID-19 exposures reported at time of study enrollment and sample collection. | | | | | | | |
| --- | --- | --- | --- | --- | --- | --- | --- |
| **Patient** | **Age** | **Gender** | **DPSO**† | **Pre-existing Conditions** | **Symptoms at Enrollment** | **Potential Exposure**‡ | **Travel**§ |
| 1 | 53 | female | 8 | hypertension,  sleep apnea | none | work | -- |
| 2 | 49 | female | -- | chronic respiratory issues, diabetes | runny nose | work | -- |
| 3 | 29 | female | 2 | none | cough, fever,  body ache | travel | domestic |
| 4 | 31 | female | 3 | none | cough, sore throat, fatigue, headache, fever, body ache, loss of taste | travel | domestic, international |
| 5 | 47 | female | 3 | hypertension | cough, headache, fever, diarrhea, high resting heart rate | unknown | -- |
| 6 | 48 | female | 3 | none | cough, sore throat, runny nose, fatigue, headache, diarrhea, body ache | travel | international |
| 7 | 38 | Male | 2 | back pain | cough, difficulty breathing, sore throat, runny nose, fatigue, headache, body ache | unknown | -- |
| 8 | 36 | female | 3 | hypothyroidism | sore throat, runny nose,  fatigue, body ache | work | -- |

†DPSO, days post symptom onset ‡Potential exposure to COVID-19 positive patient in preceding 2 months**,** § Travel in the past month
